# The neuromechanical of Beta-band corticomuscular coupling within the human motor system

**DOI:** 10.3389/fnins.2024.1441002

**Published:** 2024-08-15

**Authors:** Jiazheng Peng, Talifu Zikereya, Zhongshu Shao, Kaixuan Shi

**Affiliations:** Physical Education Department, China University of Geosciences Beijing, Beijing, China

**Keywords:** beta oscillation, brain-muscle, corticomuscular coupling, motor system, training status

## Abstract

Beta-band activity in the sensorimotor cortex is considered a potential biomarker for evaluating motor functions. The intricate connection between the brain and muscle (corticomuscular coherence), especially in beta band, was found to be modulated by multiple motor demands. This coherence also showed abnormality in motion-related disorders. However, although there has been a substantial accumulation of experimental evidence, the neural mechanisms underlie corticomuscular coupling in beta band are not yet fully clear, and some are still a matter of controversy. In this review, we summarized the findings on the impact of Beta-band corticomuscular coherence to multiple conditions (sports, exercise training, injury recovery, human functional restoration, neurodegenerative diseases, age-related changes, cognitive functions, pain and fatigue, and clinical applications), and pointed out several future directions for the scientific questions currently unsolved. In conclusion, an in-depth study of Beta-band corticomuscular coupling not only elucidates the neural mechanisms of motor control but also offers new insights and methodologies for the diagnosis and treatment of motor rehabilitation and related disorders. Understanding these mechanisms can lead to personalized neuromodulation strategies and real-time neurofeedback systems, optimizing interventions based on individual neurophysiological profiles. This personalized approach has the potential to significantly improve therapeutic outcomes and athletic performance by addressing the unique needs of each individual.

## 1 Introduction

Beta-band corticomuscular coherence (Beta-CMC) is a crucial aspect of sensorimotor integration, reflecting the interaction between the brain and muscles during movement. This introduction provides a focused overview of Beta-CMC, emphasizing its significance and relevance to motor control and sensorimotor functions.

Neural oscillations, particularly within the Beta-frequency band (12–30 Hz), are prominent in sensorimotor-related cortical and subcortical regions (Whittington et al., 2000; Kilavik et al., 2013). These oscillations are key features of neural activity and can be measured non-invasively in humans (Sherman et al., 2016; Wang et al., 2023). Beta-CMC, the coherence of Beta-band activity between the brain and muscles, is observed during isometric output and varies with the regulation of force and task precision (Koelewijn et al., 2008; Davis et al., 2012; Heinrichs-Graham et al., 2017). Beta-CMC is integral to motor planning, execution, and regulation (Kristeva et al., 2007; Mehrkanoon et al., 2014). It is modulated by peripheral inputs, highlighting the complex relationship between the brain and muscles (Riddle and Baker, 2005; Witham et al., 2011; Budini et al., 2014; Mehrkanoon et al., 2014). Understanding Beta-CMC provides insights into motor skill learning, control, and functional recovery in motor system disorders (Choi et al., 2020).

Understanding the interplay between neural oscillations and sensorimotor systems is pivotal for deciphering human motor control and its dysfunctions. Variations in research paradigms have so far obscured the clear delineation of this relationship, with the complexity of interactions between the cerebral cortex and motor systems still largely elusive (Khanna and Carmena, 2017).

This review aims to summarize findings on the dynamics of Beta-band oscillations and Beta-CMC in the sensorimotor system, focusing on their role in corticomuscular coupling and modulation during different motor phases and conditions. By integrating multisensory information, this review seeks to understand Beta-oscillations in motor control under both normal and pathological states. It will discuss recent research on pharmacological approaches and advanced brain stimulation techniques to uncover the mechanisms of Beta-band activity during sensorimotor tasks (Barone and Rossiter, 2021). The outcomes of this review aim to enhance our understanding of sensorimotor dysfunctions, leading to more precise and effective therapeutic interventions. This research benefits not only those with motor disorders but also athletes, offering insights to improve training and rehabilitation. Ultimately, this work promises to revolutionize neurology and rehabilitation treatments, benefiting patients and athletes alike by bridging clinical and performance contexts.

## 2 Band origin and mechanisms of neural oscillations in the beta frequency band

Beta oscillations, typically ranging from 13 to 30 Hz, are observed in numerous perceptual, cognitive, and motor processes (Brovelli et al., 2004; Witham et al., 2007). These oscillations are involved in diverse behavioral paradigms, including sensorimotor integration, coordination, idle-state processing, motor preparation, and attention. Given the intricate nature of Beta-oscillation activity, their origins are likely rooted in complex and varied mechanisms (Pfurtscheller et al., 1997; Kilavik et al., 2013; Shin et al., 2017; Spitzer and Haegens, 2017; Betti et al., 2021).

### 2.1 Distribution of beta oscillations across key brain regions

Beta-band neural oscillations are predominantly found in brain regions associated with the sensorimotor system, notably in the precentral gyrus (Hari and Salmelin, 1997), supplementary motor area, cingulate cortex, and dorsolateral prefrontal cortex (Sochurková et al., 2006). These oscillations are also observed in the sensorimotor and premotor cortices, parietal lobes, and cerebellum (Fujioka et al., 2015), basal ganglia as well as in the various muscle locations (Baker, 2007; De Marchis et al., 2015; Rana et al., 2015; Reyes et al., 2017), the spinal cord (as evidenced in primates) (Oya et al., 2020), the dorsal root ganglia (Baker et al., 2006), and peripheral motor units (Blenkinsop et al., 2017). Beta oscillations are involved in all motor control-related systems, indicating their significant role in the overall functionality of the motor system.

**Figure 1:**
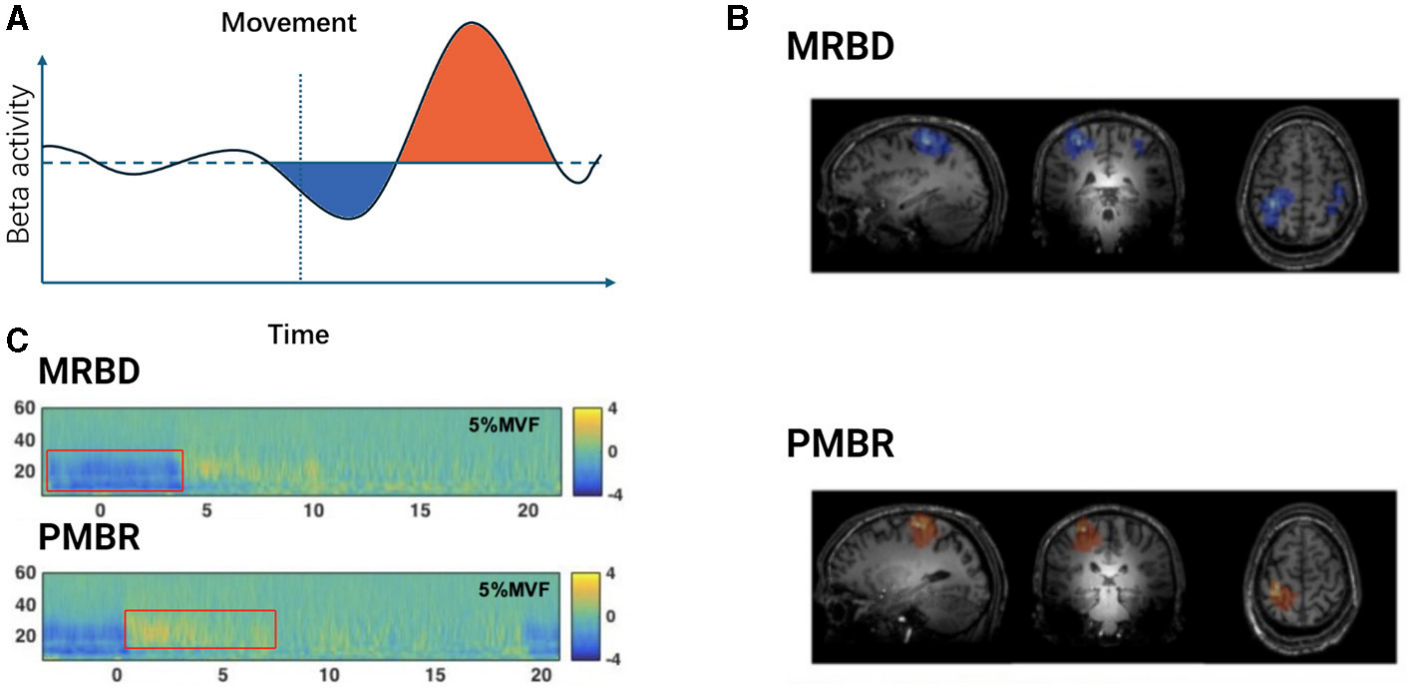
Temporal dynamics of beta oscillations during movement execution. **(A)** Schematic representation of Beta activity dynamics during motor tasks. The light blue shaded area indicates the period of Movement-Related Beta Desynchronization (MRBD) occurring before and during movement execution. The light purple shaded area represents the Post-Movement Beta Rebound (PMBR), which increases swiftly after task completion and slowly returns to baseline levels. **(B)** Spatial features of MRBD (top) and PMBR (bottom) in an individual participant. Adapted from Seedat et al. (2020). **(C)** Time-frequency spectra extracted at peak locations during a 5% Maximum Voluntary Force (MVF) isometric wrist flexion task, depicting MRBD (top) and PMBR (bottom). Adapted from Fry et al. (2016).

Typically, beta oscillations are present during stable motor states and decrease during movement. The variations in beta oscillations during motor-related neurophysiological processes are often attributed to the synchronized activity of neurons in specific local areas of the motor cortex (Espenhahn et al., 2017; Barone and Rossiter, 2021). This phenomenon has been observed in multiple studies, where motor-related beta decrease (MRBD) and post-movement beta rebound (PMBR) are considered classic examples of event-related desynchronization/synchronization (ERD/S) (Stancák and Pfurtscheller, 1995; Byrne et al., 2017). Figure 1 illustrates PMBR/MRBD. These phenomena reflect the complex neural regulatory mechanisms involved in motor execution and control. The stability of these changes appears consistent across different effectors, types of movement, speeds, complexities of movement, and age groups (Kilavik et al., 2013).

### 2.2 Regional characteristics and mechanisms of beta oscillations in EEG

Beta oscillations in EEG signals can be categorized into Rolandic beta and Frontal beta, each exhibiting distinct regional characteristics and functional associations. Frontal beta rhythm generally displays maximal power in the frontal lobe areas and is associated with cognitive tasks such as stimulus evaluation and decision-making (Stoll et al., 2015; Schmidt et al., 2019). In contrast, Rolandic beta rhythm exhibits its greatest power in the sensorimotor regions and is linked with tasks involving motor imagery, motor preparation, and motor execution (Pfurtscheller and Solis-Escalante, 2009; Brinkman et al., 2014; Nijhuis et al., 2021). Often termed the Rolandic beta indicates a “resting state” of brain activity (Pfurtscheller et al., 1996a; Fairhall et al., 2007; Ritter et al., 2009), where its presence during rest negatively correlates with heart rate variability (Triggiani et al., 2016). This rhythm becomes particularly active during motor preparation and execution, showing a negative correlation with the timing of motor decisions (Jo et al., 2016).

### 2.3 Types and mechanisms of beta oscillations

Initial studies identified two main types of Beta-oscillations: one associated with the μ-rhythm, ~22–24 Hz, showing desynchronization (Event-Related Desynchronization, ERD) before and during movement, and slow synchronization recovery post-movement. The other type, post-movement beta synchronization (PMBS), starts to desynchronize shortly before movement and rapidly resynchronizes afterward, lasting about 1–2 s, predominantly within the 12–26 Hz frequency range and showing contralateral dominance (Pfurtscheller, 1981; Pfurtscheller et al., 1997). Differential Beta frequencies in cortical hand and foot areas suggest variations in neural network structures and interconnectivity across specific sensorimotor cortical regions, indicating that different Beta oscillations may be specific to different motor areas (Pfurtscheller et al., 2000; Neuper and Pfurtscheller, 2001). Current research often divides Beta-band oscillations into lower and higher frequency bands, using 20 Hz as a demarcation line (Engel and Fries, 2010; Saleh et al., 2010; Schmidt et al., 2019).

Beta oscillations, particularly Beta1 (≈15 Hz) oscillations, were first identified in experimental and modeling studies within the association area of the cerebral cortex in rats (Kramer et al., 2008). These rhythms are thought to form through the interaction and temporal coordination between deep and superficial cortical cells, becoming prominent after transient excitatory (sensory) inputs are removed (Roopun et al., 2008). The sustainability of this rhythm does not depend on synaptic plasticity but is determined by the cells' response to inhibitory rebound, allowing these assemblies to sustain themselves by responding to both familiar and novel stimuli (Whittington et al., 2000).

Beta2 (20–30 Hz) oscillations are thought to originate within non-synaptic networks of layer V pyramidal cells, which contribute to the corticospinal tract. These oscillations rely on gap junction coupling and can persist even when layer IV is removed, suggesting they do not depend on apical dendritic electrogenesis (Roopun et al., 2006). M-type K+ currents are believed to determine the oscillatory period, suggesting that cortical network oscillations under normal conditions may predominantly arise from non-synaptic mechanisms.

Furthermore, the experimental models illustrate that beta activity can facilitate inter-layer and intra-layer interactions, where groups of neurons synchronized within the beta band can coexist with other cell groups (Kilavik et al., 2013). Beta oscillations have complex generation mechanisms and unique anti-dynamics properties (Donoghue et al., 2022), allowing them to persist long after excitatory inputs have decayed (Kopell et al., 2011). This diverse neural oscillatory rhythm is likely closely related to broader endogenous top-down processing and sensorimotor integration, as discussed by Barone and Rossiter (2021). Additionally, in motor control processes, there is a quantifiable relationship between local concentrations of gamma-aminobutyric acid (GABA) and beta amplitude (Hall et al., 2011; Muthukumaraswamy et al., 2013; Rossiter et al., 2014), with high-frequency beta2 oscillations possibly playing a significant role. The generation of sensorimotor beta oscillations is thought to be regulated by phase-locked GABA-mediated interneuronal inputs associated with the activity of layer V pyramidal cells (Baker, 2007; Gaetz et al., 2011). Computational neural models suggest that layer V pyramidal cells exhibit alternating depolarization and hyperpolarization, an interaction that triggers sensorimotor beta oscillations (Baker, 2007; Bhatt et al., 2016; Wischnewski et al., 2022). Therefore, subsequent research often links changes in motor cortex beta oscillations with variations in GABA, and associated changes in cortical inhibition and plasticity.

Firstly, beta oscillations play a crucial regulatory role in motor control by coordinating neuronal synchronization, which ensures the precise transmission and execution of motor commands. Secondly, the relationship between local gamma-aminobutyric acid (GABA) concentrations and beta amplitude indicates that GABA significantly influences motor function, directly affecting motor control and coordination. Additionally, beta oscillations are involved in sensorimotor integration, suggesting that the brain utilizes these oscillations to coordinate sensory input and motor output, thereby ensuring the accuracy and fluidity of movements. In terms of neural plasticity, beta oscillations are associated with changes in cortical inhibition and plasticity, which are essential for motor learning and adaptation, particularly in the acquisition of new motor skills. Finally, computational neural models propose that alternating depolarization and hyperpolarization of layer V pyramidal cells trigger sensorimotor beta oscillations. This mechanism provides a theoretical foundation for understanding the neural processes underlying motor control and may contribute to the development of novel treatments for motor disorders.

### 2.4 Mechanisms of abnormal beta oscillations

In Parkinson's disease, beta frequency oscillations in the basal ganglia and cortex may originate from inhibitory interactions between medium spiny neurons in the striatum. McCarthy et al. (2011) found through mathematical modeling and experimental observations that amplification of striatal network dynamics could enhance beta frequency oscillations. When a cholinergic agonist was injected into the striatum of normal, awake animals, significant beta frequency oscillations were observed, aligning with model predictions. These oscillations were linked to synaptic GABAa currents and intracellular M currents, promoting collective beta frequency oscillations (Shimono et al., 2000; Deffains and Bergman, 2015; Kondabolu et al., 2016).

**Figure 2:**
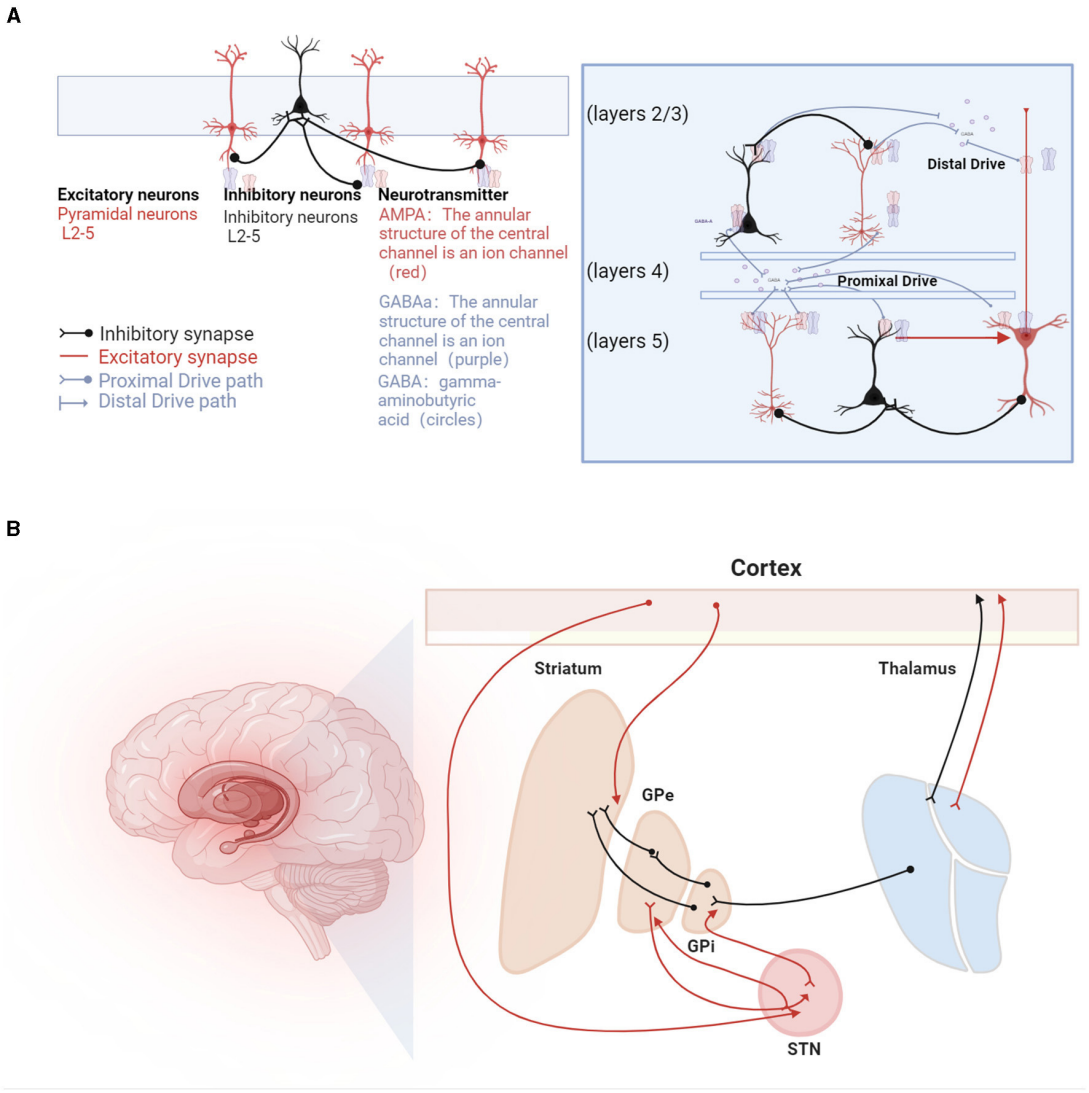
Beta oscillations in the sensorimotor system. **(A)** Illustration of the mechanisms underlying beta oscillation generation. On the left, beta oscillations are depicted as arising from recurrent interactions within deep cortical layers (Lacey et al., 2014), involving pyramidal neurons (represented as red triangles) and interneurons (depicted as green circles). GABA (gamma-aminobutyric acid) is shown as circles, with GABAa receptors forming the annular structure of a central ion channel (purple), and AMPA receptors, also forming a central ion channel structure (red), primarily facilitate rapid excitatory transmission, while GABAa receptors are mainly responsible for rapid inhibitory transmission. At the center, a laminar model displays beta generation facilitated by pyramidal neurons located in both supragranular (layers 2/3) and infragranular layers (layer 5), influenced by dual external excitatory inputs, predominantly from the thalamus (Sherman et al., 2016). Additionally, beta bursts are generated by a model incorporating a broad proximal excitatory synaptic drive synchronized with a strong distal synaptic drive (Bonaiuto et al., 2021). **(B)** Hypotheses regarding the generation of beta rhythms in the basal ganglia pathways, involving inhibitory and excitatory circuits: (1) STN-GPe Rhythm Hypothesis: Beta oscillations originate from the network interactions between the subthalamic nucleus (STN) and the external globus pallidus (GPe). (2) Cortical Origin Hypothesis: In Parkinson's disease (PD) patients, beta oscillations are thought to originate from the cortical-basal ganglia-thalamocortical loop. (3) Striatal Origin Theory: Enhanced beta rhythms result from increased inhibitory interactions among striatal neurons. (4) Integrated Neural Circuit Changes Theory: Excessive beta rhythms are hypothesized to arise from a composite effect of inherent neuronal properties within the cortical-basal ganglia-thalamocortical loop and its associated circuits (not listed).

The mechanisms underlying beta oscillations are not fully understood, with hypotheses proposing both cortical and subcortical origins. Cortical genesis theories, supported by *in vitro* studies, suggest potential pathways involving transmission from superficial to deep layers of pyramidal cells (Bollimunta et al., 2008). These studies indicate that the activation of deep pyramidal cell layers or synchronized hyperpolarization across layers can induce Beta-oscillations (Weiler et al., 2008; Bhatt et al., 2016). Biophysical modeling predicts that high-amplitude beta bursts in human motor and sensory cortices may originate from temporally aligned excitatory synaptic drives across deep and superficial layers (Bonaiuto et al., 2021). Different mechanisms of beta generation are illustrated in Figure 2.

Subcortical theories focus on the basal ganglia, particularly the STN-GPe loop within the striato-thalamo-cortical circuitry (Holgado et al., 2010). Chronic dopamine depletion in Parkinson's disease may reorganize the cortico-basal ganglia-thalamo-cortical (CBGT) circuit. However, these models, which involve changes in connections from the cortex to subthalamic nuclei and from the STN to the external globus pallidus, have not yielded unanimous results. Liu et al. (2020) proposed a dual-oscillator system encompassing the BG-Th network and the cortex, capable of generating high or low-frequency Beta1 or Beta2 oscillations depending on the structure of the oscillators, suggesting a possible theory for the multiple origins of Beta-oscillations.

## 3 Functional roles of beta oscillations

Neural oscillations are a hallmark of brain network information processing (Han et al., 2021a,b), yet a consistent one-to-one mapping between these oscillations and brain network activities does not seem to exist (Doelling and Assaneo, 2021; Lundqvist and Wutz, 2022). Although many studies have observed correlations between neural activity and other physiological signals, beta oscillations appear to be specifically related to task-relevant information (Spitzer and Haegens, 2017). This includes the generation of motor goals (Fischer et al., 2017), maintenance and monitoring of tasks and states (Shin et al., 2017; Little et al., 2018), and learning and adaptation to motor-related errors (Pollok et al., 2014; Wang et al., 2019). Complex brain network activities in different states affect the amplitude, frequency, timing, and distribution of beta oscillations (Schmidt et al., 2019).

### 3.1 Functional roles of beta oscillations and task-specific information processing

Historically, some researchers believed that beta oscillations might reflect a concept where the motor system is in an “idling” state (Pfurtscheller et al., 1996a; Kilavik et al., 2013), representing the processing of motor-related sensory information (Salmelin and Hari, 1994). However, increasing evidence suggests that the “idling” concept does not fully explain the function of beta oscillations in sensorimotor activities. Instead, beta oscillations are likely involved in maintaining the current sensorimotor or cognitive state (Pfurtscheller et al., 1996b; Fairhall et al., 2007), rather than merely reflecting the motor system's idle state. For instance, Cassim's study showed that ischemia-induced reduction in incoming sensory feedback led to the disappearance of beta oscillations, broadening our understanding of their role in the sensorimotor system beyond merely “idling” (Cassim et al., 2001). Beta oscillations not only facilitate the stabilization of movements but also influence the generation of new movements (Engel and Fries, 2010). They are more pronounced in processing unattended stimuli and during motor-related anticipatory processes, such as when compensating for expected disturbances or maintaining a specific motor state (Caetano et al., 2007). This reflects top-down control signals used to suppress irrelevant information or disturbances and regulate the motor system (Gilbertson et al., 2005).

#### 3.1.1 Gating mechanisms of beta oscillations in sensorimotor processing

As proposed by Jensen and Mazaheri, the “gating theory” suggests that information is transmitted by functionally blocking pathways unrelated to the task at hand. Through inhibitory gating, beta oscillations primarily involve gating in the somatosensory cortex by suppressing upcoming sensorimotor transformations across different cortical activity bands (Jensen and Mazaheri, 2010; Talsma et al., 2010). Stevenson et al. viewed beta oscillations as a form of local cortical gating aimed at facilitating complex neural activities, such as information processing. In some circumstances, local neurons may reduce beta amplitude to accommodate more complex neural activities, as observed by Schulz et al., where motor-related beta suppression (ERD) coincided with enhanced muscle coupling within the alpha band, and beta rebound (ERS) was associated with reduced muscle coupling. This confirms that enhancements in Beta-band oscillations reflect stabilization or inhibitory mechanisms of the motor system, hindering the activation or selection of new motor behaviors (Schulz et al., 2013). Consistent findings have been observed in human magnetoencephalography during attention tasks and in local field potentials in mice performing execution detection tasks, indicating that an increase in beta oscillations signals reduced efficiency in information transmission (Shin et al., 2017). This also explains why extensive studies have noted motor-related beta decrease (MRBD) before movement, and post-movement beta rebound (PMBR) associated with suppressed somatosensory processing and sensory input to motor actions (Stevenson et al., 2011; Limanowski et al., 2020). According to this hypothesis, more complex neural activities, such as motor planning and execution, monopolize neural resources. Similar phenomena occur during imagined and observed movements (Kilavik et al., 2013; Buchholz et al., 2014).

#### 3.1.2 Cognitive processing and beta oscillations in motor actions

Motor processes are dynamically regulated through coordination between cognitive processing in the brain and the motor system (Brisswalter et al., 2002). Complex motor actions involve cognitive decisions and judgments, and brain regions associated with these functions have also been reported to exhibit beta oscillation activity (Koelewijn et al., 2008; Alayrangues et al., 2019). Lundqvist et al. (2011, 2016, 2018) reported an increase in theta and gamma power with increased working memory load, alongside a decrease in alpha/beta power, indicating the involvement of beta oscillations in cognitive functions, particularly working memory. Experiments on motor anticipation and selection of specific objects have revealed the potential role of beta oscillations in flexibly controlling working memory (Lundqvist et al., 2018). The functionality of beta oscillations related to working memory in the prefrontal cortex (PFC) has been extensively discussed, highlighting their significance in cognitive control mechanisms (Schmidt et al., 2019).

It is noteworthy that, during sustained isometric contraction tasks, the short “burst” characteristics of neural oscillations and connectivity between the brain and muscles have been observed (Echeverria-Altuna et al., 2022). Analyzing neural oscillations as a series of transient burst events rather than continuous oscillatory activities offers an exciting new perspective (van Ede et al., 2018; Doelling and Assaneo, 2021; Rayson et al., 2023). The intermittent, transient, high-power burst events observed during various neural activities are also significant; analyzing these events across different dimensions of time, spectrum, and space presents challenges and is crucial for accurately describing event characteristics and revealing their interactions (Zich et al., 2020; Doelling and Assaneo, 2021). This approach enhances our understanding of brain dynamics across different tasks and cognitive states, enabling the capture of non-periodic features of the brain that aid in elucidating its role in various cognitive functions such as attention, memory, and consciousness. This advancement further propels our understanding of neural oscillations. Further insights into beta oscillations during the stages of information encoding, retrieval, and selective deletion have been provided by previous studies. Cross-regional interaction studies have highlighted the crucial role of beta oscillations in coordinating brain networks during both task execution and resting states, with additional discussions on their involvement in cognitive processing (Lundqvist et al., 2024). Investigating the neural circuit origins of beta bursts, their shared mechanisms in cognition and action stopping, and the potential of beta burst analysis to enhance the diagnosis and treatment of neurological diseases remain pivotal areas for future research.

The activity in the beta frequency band holds significant biomarker potential within the sensorimotor system, particularly in pathological contexts. Given the complex composition of the motor system, the effects of different motor parameters on beta oscillations and their role in brain-muscle communication require further investigation and validation. The modulation of brain oscillation power may be closely related to the degree of spike synchronization and the balance between excitatory and inhibitory signals within the neuronal network (Buzsáki and Draguhn, 2004; Han et al., 2023b). Therefore, oscillations at different frequencies might reflect distinct states of neuronal clusters or networks. To establish a strong link between oscillations and behavior, it is essential to explore how these oscillations reflect and drive underlying neural activity (Kirschstein and Köhling, 2009). This deeper understanding will not only enhance our comprehension of the functional dynamics within the sensorimotor system but also improve our ability to effectively address motor system dysfunctions.

## 4 Beta oscillations in the context of corticomuscular coherence

### 4.1 Characteristics and functions of beta oscillations within the CMC context

The study of functional connectivity between the cerebral cortex and muscles during motor states effectively models changes in brain networks (Schulz et al., 2013). Motor commands issued from the motor cortex lead to muscle contractions through efferent motor pathways and are modulated by afferent somatosensory pathways (Schomburg, 1990; Rijntjes et al., 1999). The functional coupling of electrophysiological signals between the cortex and muscles, known as corticomuscular coupling, is typically measured using corticomuscular coherence (CMC). serves as a biomarker for corticomuscular connectivity, providing insights into cortical control of muscle function (Fauvet et al., 2021).

**Figure 3:**
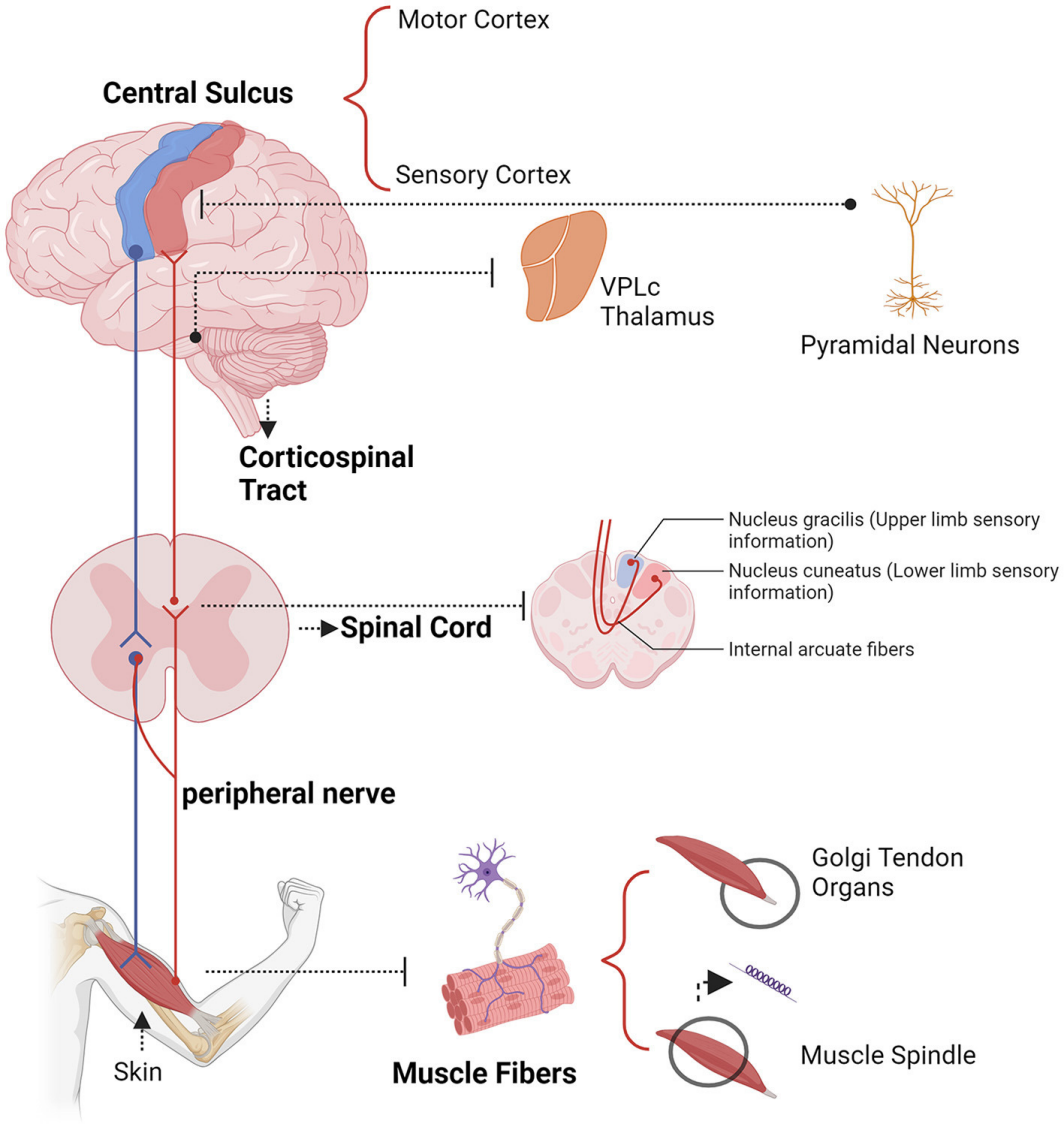
Example descending (blue) and ascending (yellow) pathways which could mediate corticomuscular coherence.

Electrophysiological techniques such as EEG, MEG, ECoG, and intracranial electrode recordings offer millisecond-level temporal resolution, advancing our understanding of neural oscillations (Kirschstein and Köhling, 2009; Baillet, 2017; Han et al., 2022, 2023a; Lin et al., 2024; Wang B. et al., 2024). These technologies enable real-time tracking of neural signals, revealing the dynamics of neural activity (Lundqvist and Wutz, 2022). CMC reflects the activity of sensorimotor networks during dynamic movements and isometric contractions, useful for diagnosing and rehabilitating movement disorders (Airaksinen et al., 2015; Liu et al., 2019a). A key challenge in neurophysiology is understanding the synchronization between EEG and EMG signals, which constitutes CMC (Kasuga et al., 2018). Conway et al. (1995, 2004) discovered significant beta band coherence (13–35 Hz) between cortical activity and EMG of contralateral hand muscles, indicating synchronized cortical neuronal activity relates to motor unit firing. CMC is most commonly observed during isometric contractions and is associated with stable force output, primarily in the beta frequency band (15–30 Hz). This coupling of information between the cortex and muscles, predominantly found in the beta frequency band (15–30 Hz) (Mima et al., 2002; Engel and Fries, 2010; Mehrkanoon et al., 2014). The pathways mediating corticomuscular coupling are illustrated in Figure 3.

### 4.2 Coherence measurement and analytical techniques in beta-CMC

Coherence measures the linear connection between two signals in the frequency domain. In CMC, it assesses the synchrony between brain activity, recorded via electroencephalography (EEG), and muscle activity, recorded via electromyography (EMG). The coherence between EEG and EMG signals is calculated using the normalized cross-spectrum density:


$$\begin{array}{l} {\text{Coh}}_{\text{EEG},\text{EMG}}\left(\text{f}\right)=\frac{|P_{EEG,EMG}\left(f\right){|}^{2}}{{\text{P}}_{\text{EEG}}\left(\text{f}\right)\cdot {\text{P}}_{\text{EMG}}\left(\text{f}\right)} \end{array}$$

where (*P*_*EEG, EMG*_(*f*)) is the cross-power spectrum of the EEG and EMG signals at frequency (*f*), and (*P*_*EEG*_(*f*)) and (*P*_*EMG*_(*f*)) are the power spectra of the EEG and EMG signals at frequency *f*, respectively. Coherence values range from 0 to 1, with higher values indicating a stronger correlation between the two signals (Mima et al., 1999). High coherence at specific frequencies suggests robust neural communication from the cortex to the muscles, indicating effective corticospinal pathways.

CMC is crucial for understanding motor control mechanisms, particularly in movement disorders and rehabilitation strategies. It dynamically adjusts with muscle contraction patterns, reflecting top-down motor information transmission (Baker et al., 1997; Brown et al., 1998; Mima et al., 1999; Ushiyama et al., 2012; Boonstra, 2013).

Initial methods for CMC analysis involved Fourier coherence and partial directed coherence (Grosse et al., 2002; Schelter et al., 2006; Yao et al., 2007). Both methods handle non-stationary signals; however, wavelet coherence, with its fixed window size, adapts better to the frequency of oscillatory signals, providing more accurate results. Partial directed coherence evaluates the direction of neural information flow, offering insights into the functional connection between cortical and muscular signals. Wavelet coherence has become widely adopted due to its ability to handle non-stationary signals and provide time-frequency localized information (Yang et al., 2010; Xi et al., 2021).

To further advance CMC analysis, researchers have explored various dimensions such as local frequency bands, cross-frequency coupling, time delays, and multiscale characteristics. Functional corticomuscular coupling (FCMC), essentially another term for CMC, was first introduced by Yang et al. (2009). Functional corticomuscular coupling (FCMC) probes multi-level information communication in the sensorimotor system (Ibáñez et al., 2021). Traditional methods like canonical coherence (caCOH) have been used to measure FCMC between multivariate signals at a single scale (Vidaurre et al., 2019).

Recent advancements propose multiscale canonical coherence (MS-caCOH) to disentangle complex multi-layer information across multiple scales, demonstrating enhanced coupling detection and lower pattern recovery errors (Sun et al., 2024). Similarly, composite multiscale coherence (CMSC) models explore FCMC in motor control systems, showing stability at high time scales and capturing multiscale characteristics with higher coherence in alpha and beta bands (Chen et al., 2023). These methods extend FCMC research by offering robust and detailed multiscale interaction analysis. In addition to these methods, advanced techniques such as multiscale transfer entropy (MSTSE) have been introduced to describe multi-layer neural information transfer between coupling signals (Xi et al., 2022). MSTSE is more robust and effective in detecting coupling properties compared to single-scale methods, allowing for the analysis of FCMC at various scales and frequencies, providing a comprehensive understanding of the multi-scale characteristics of FCMC (Sun et al., 2024).

Studies have also revealed nonlinear properties in the sensorimotor control loop. Linear coupling is primarily driven by descending motor pathways, while afferent sensory feedback contributes to nonlinear coupling patterns (Myers et al., 2003; Yang et al., 2018; Liang et al., 2020). The integration of nonlinear coupling algorithms and advanced modeling techniques continues to enhance our understanding of the neural mechanisms underlying CMC, facilitating the identification of factors affecting CMC.

There is ongoing debate regarding the rectification of EMG in CMC calculations (Yoshitake and Shinohara, 2013; McClelland et al., 2014). While rectification is often used to maximize information about action potential timing and suppress information related to motor unit action potential (MUAP) shape, some studies suggest that it does not enhance the detection of CMC. Rectification can distort the EMG spectrum and obscure genuine CMC detection in some cases (Neto et al., 2010). Therefore, it is argued that coherence analysis should be performed using unrectified EMG to avoid these issues (McClelland et al., 2012). This perspective highlights the need for careful consideration of preprocessing steps in CMC analysis to ensure accurate and reliable results.

### 4.3 Beta-CMC in different motor tasks

#### 4.3.1 Beta-CMC in stable motor states

In this section, the term “stable motor states” refers specifically to motor conditions designed to minimize interference from electromyographic (EMG) noise. These stable conditions include single-joint movements, isometric contractions, and other controlled motor tasks that reduce muscle activity artifacts. By focusing on these stable states, researchers can better isolate and study the underlying neural mechanisms of Beta-band corticomuscular coherence (CMC) without the confounding effects of more complex, multi-joint movements. This approach ensures that the observed CMC reflects true corticospinal communication rather than extraneous muscle activity.

Beta-CMC disappears before the start of a movement and increases during isometric contractions with low-level static force adjustments, potentially stabilizing corticospinal information exchange (Chakarov et al., 2009). During dynamic force output, it is replaced by transient synchronization in the alpha and gamma bands, with phase synchronization in different frequency bands indicating incoming and outgoing corticospinal interactions (Mehrkanoon et al., 2014). Changes in Beta-CMC under different motor tasks or states are summarized in Table 1. Bottom-up Beta-band activity may facilitate steady isometric contractions by effectively transmitting sensory feedback from the finger muscles to the sensorimotor cortex (Lim et al., 2014). Changes in CMC phase induced by cooling the arm (Riddle and Baker, 2005) and ischemia-induced reductions in afferent nerve capability (Pohja and Salenius, 2003) have demonstrated the role of incoming peripheral sensory signals in sensorimotor communication. Thus, CMC is considered to be regulated by top-down motor commands and feedback signals from proprioceptors, which also modulate this (Budini et al., 2014). Interestingly, pharmacological studies have shown that enhancements in EEG signals by benzodiazepines do not modulate the amplitude of CMC (Baker and Baker, 2003), while various types of GABAergic medications produce diverse modulations of cortical activity and CMC amplitude (Barone and Rossiter, 2021).

**Table 1:** Summary of Beta-band corticomuscular coherence (Beta-CMC) changes under different conditions.

**Condition**	**Beta-CMC changes**	**References**
**Stable motor states**		
Isometric contraction tasks	Significant Beta-CMC observed, indicating the cerebral cortex's role in maintaining balance and stability	Jacobs et al., 2015; Liu et al., 2019b
Isokinetic contraction tasks	CMC differences disappear, suggesting a shift from feedforward to feedback regulation in motor control	Liu et al., 2019b; Suzuki and Ushiyama, 2020
**Different motor states**		
Large amplitude movement states	Few studies due to the subtle and unstable nature of EEG signals, limiting exploration of limb CMC	Gennaro and de Bruin, 2020; Kenville et al., 2020; Xi et al., 2022
Treadmill walking	Significant beta and gamma band coherence between EEG and EMG signals from the tibialis anterior and soleus muscles during stance and propulsion phases	Jensen et al., 2019; Gennaro and de Bruin, 2020
Ground walking during double-support phase	Higher Beta-CMC observed, with EEG signals preceding EMG signals, indicating cortical activity leads muscle activity	Roeder et al., 2018
Periodic bilateral ankle movements	Increased coherence near 20 Hz, primarily in brain regions directly controlling the tibialis anterior and soleus muscles; coherence enhanced with external rhythmic guidance	Yoshida et al., 2017
Different task conditions and force levels	CMC exhibits different patterns; decreases with increased contraction intensity during intermittent elastic tasks, but differences disappear in sustained isometric tasks	Suzuki and Ushiyama, 2020
**Sensorimotor integration**		
External sensory feedback (visual, auditory)	Changes in sensory feedback (e.g., reduced visual feedback) lower Beta-CMC peak frequency, indicating the influence of sensory inputs on CMC	Chung et al., 2017; Chen et al., 2023

What is the connection between widely observed Beta-CMC and movement during stable motor processes? Prior to the initiation of movement, a reduction in beta power is associated with faster autonomous movements (Gilbertson et al., 2005; Shin et al., 2017). When the amplitude of Beta-CMC increases, the generation of new movements is delayed (Matsuya et al., 2013), and prolonged elevated beta oscillations have been observed in Parkinson's disease, associated with difficulties in initiating and controlling movements (Brown, 2006; Asadi et al., 2022).

These findings suggest that the mechanisms underlying CMC are complex and not merely a simple unidirectional transmission phenomenon. Instead, they involve a complex interplay of motor commands and sensory feedback. This coherence relates to sensorimotor integration functions (Kilavik et al., 2013), indicating a comprehensive and mutually regulatory relationship between motor commands and sensory feedback (Witham et al., 2011).

#### 4.3.2 Beta-band CMC across dynamic motor states

Significant Beta-CMC has been observed in human standing tasks, highlighting the cerebral cortex's role in maintaining balance and responding to changes in mechanical and sensory conditions (Jacobs et al., 2015). However, due to the subtle and unstable nature of EEG signals, few studies have explored limb CMC during large-amplitude movements (Gennaro and De Bruin, 2018; Zhao et al., 2022a). This section focuses on the role of Beta-Band CMC across various dynamic motor states, emphasizing the impact of large-amplitude movements on EEG signals.

Jensen et al. (2019) investigated CMC during treadmill walking, finding significant beta and gamma band coherence between EEG and EMG signals from the tibialis anterior and soleus muscles during the stance and propulsion phases of gait. Directional analysis showed EEG activity led EMG activity during the support phase and forward propulsion (Jensen et al., 2019). Similarly, Roeder et al. (2018) reported higher CMC during the double-support phase of ground walking, with EEG signals preceding EMG signals. These findings suggest a crucial role for Beta-CMC in coordinating complex motor tasks.

In neuromuscular coupling research during gait, involving both healthy individuals and those with neuromuscular or nervous system diseases, the synchronicity between EEG and EMG signals, defined as Neuromuscular Connectivity (NMC), has been explored (Zhao et al., 2022b). While NMC holds significant potential for assessing brain-muscle interactions, there is a need for standardizing research methodologies to enhance comparability and reproducibility (Zhao et al., 2022b; Seynaeve et al., 2024).

Yoshida et al. found that during periodic bilateral ankle movements, brain regions controlling movement showed increased coherence near 20 Hz with the tibialis anterior and soleus muscles. This coordination intensified with external rhythmic guidance, enhancing focus on movement (Yoshida et al., 2017). Beta-band activity dynamically adjusts to motor task demands, indicating that neural synchronization and connectivity may involve brief “bursts” rather than continuous states (Mirzaei et al., 2017).

The generation of beta oscillations varies with different activities. Khanna and Carmena noted that beta activity is commonly produced in the striatum during significant external stimuli, adjusting internally planned actions. During static isometric contractions, beta activity relates to autonomous contractions, involving pyramidal tract neurons discharging in the beta range, which increases motor neuron activity and muscle force production (Khanna and Carmena, 2017). Thus, beta oscillations are linked to action planning, muscle coordination, and force production, reflecting how the brain regulates these processes (Iwama et al., 2022), and physiologically reflect how the brain regulates coordination among different muscles.

While these findings provide valuable insights, there are limitations. The instability of EEG signals poses challenges in studying large amplitude movements. Future research should focus on standardizing NMC methodologies and exploring new techniques to overcome these limitations. Understanding the dynamic interactions between cortical regions and muscles across various motor tasks will enhance our knowledge of sensorimotor integration and inform more precise interventions for motor disorders.

#### 4.3.3 Beta-band CMC in force control and precision movements

Beta-CMC is prominently observed during stable isometric contractions. While some studies suggest that Beta- does not significantly vary with motor parameters such as movement speed and accuracy (Kilavik et al., 2013; Dal Maso et al., 2017), other research indicates a positive correlation between Beta- amplitude and movement precision. For instance, during a pinch grip task, Beta- reflects a synergistic control strategy, integrating task-relevant motor neurons into functional units (Reyes et al., 2017).

Studies have shown a positive correlation between Beta-CMC amplitude and movement precision. Under dual-task conditions, where attentional resources are divided, CMC amplitude decreases, yet higher-frequency Beta-CMC is associated with greater precision in motor tasks (Kristeva-Feige et al., 2002; Kristeva et al., 2007).

Conversely, Johnson found that additional tasks reduce Beta-CMC, highlighting the impact of divided attention on corticomuscular coupling (Johnson et al., 2011). Further research has indicated that internal focus during tasks can decrease Beta-CMC and impair force accuracy and stability (Parr et al., 2023a). For example, when one hand is engaged in medium strength contractions, the other hand shows increased CMC due to extensive bilateral cortical connections (Zheng et al., 2016). This suggests that attentional demands significantly influence Beta-CMC.

Divekar explored differences between wrist flexors and extensors, finding that frequent use and lower perceptual difficulty of wrist flexors lead to better adaptation and lower CMC levels during isometric tasks (Divekar, 2013). Precise motor control is linked to bilateral supplementary motor area (SMA) activity, with SMA projections to the corticospinal tract becoming significant for high-precision tasks (Matsuya et al., 2013). Desmyttere's study reported that co-activation of synergistic muscles decreases Beta-CMC, while antagonist muscle activation increases it, suggesting a role in fine motor control (Desmyttere et al., 2018). Averbeck hypothesized that coherent oscillations between neurons reflect dynamic information flow, with steady-state CMC being suboptimal under unpredictable force conditions (Averbeck and Lee, 2004; Mendez-Balbuena et al., 2012).

Ushiyama found that CMC decreases with increasing contraction intensity during intermittent elastic tasks but not in sustained isometric tasks, indicating context-dependent modulation (Suzuki and Ushiyama, 2020). These findings suggest that Beta-CMC reflects a shift from feedforward to feedback regulation in motor control, influenced by factors such as force magnitude, attention, and task complexity (Lattari et al., 2010).

The information highlights the complexity and context-dependency of Beta-CMC. Beta-CMC is crucial for maintaining stable muscle force during isometric contractions, but its relationship with motor parameters like movement speed and accuracy is less consistent. Attention significantly affects Beta-CMC, with divided attention reducing its amplitude, while higher-frequency Beta-CMC is linked to greater motor precision. Beta-CMC's dynamic adjustment underscores the complexity of corticomuscular connections. It exhibits different patterns under varying task conditions and force levels. For example, Beta-CMC decreases with increased contraction intensity during intermittent elastic tasks, but this difference disappears in sustained isometric tasks. This context-dependent modulation suggests Beta-CMC reflects a shift from feedforward to feedback regulation in motor control. Additionally, sensory feedback and common inputs are crucial for CMC, requiring further experimental validation.

Future research should explore the interactions between different cortical regions and muscle groups across various motor tasks, incorporating both intermuscular coherence (IMC) and CMC to understand broader neural network dynamics. This will provide insights into how the brain controls muscle activity and adapts to different motor demands, potentially leading to more precise interventions for motor disorders.

#### 4.3.4 Beta-band CMC in sensorimotor integration

The sensorimotor cortex continuously processes dynamic stimuli from the environment, crucially regulating autonomous movements (Hohlefeld et al., 2011; Piitulainen et al., 2021). Primates can spontaneously synchronize with environmental rhythms, and these stimuli modulate Beta-CMC (Lattari et al., 2010; Piitulainen et al., 2015; Wang G. et al., 2024). This modulation occurs in areas such as the basal ganglia, cerebellum, SMA, pre-SMA, and PMC, dynamically adjusting to external stimuli (Saleh et al., 2010; Fujioka et al., 2012). Varlet et al. (2020) found that Beta-CMC plays a role in the synchronization of movements with 2 Hz audio-visual sequences, indicating its potential mechanism for movement synchronization.

Rhythmic structure perception in the brain extends beyond auditory areas to involve the sensorimotor cortex, basal ganglia, and hippocampus. During metronome listening, non-phase-locked beta oscillations synchronize across bilateral auditory cortices and motor-related areas, forming a functional sensorimotor network where beta oscillations play a key role (Haenschel et al., 2000; Abbasi and Gross, 2020; Gourévitch et al., 2020). Even in passive auditory conditions, beta oscillations dynamically configure the sensorimotor network, reflecting functional coordination between auditory and motor systems (Fujioka et al., 2012, 2015). Auditory feedback has been shown to reduce alpha spectrum in the ipsilateral sensorimotor area and beta spectrum bilaterally, decreasing Beta-CMC while enhancing motor precision (Guo et al., 2022). Similarly, optimal noise conditions improve motor accuracy and enhance motor spectral power (SP) and Beta-CMC (Trenado et al., 2014). These findings suggest that stochastic resonance enhances motor performance, consistent with increases in motor SP and CMC (Mendez-Balbuena et al., 2012; Trenado et al., 2014).

Beta-CMC is modulated by various external sensory signals. For instance, reduced visual feedback decreases the peak frequency of Beta-CMC and increases its amplitude, accompanied by a reduction in EEG Beta-band power (L'Abbate et al., 2022). Increased tactile feedback leads to right occipital cortex Beta-ERD and smaller motor errors (Lin et al., 2012). High visual gain conditions result in more pronounced Beta-band desynchronization, superior motor performance, and fewer motor errors, with enhanced connectivity between the parietal and motor cortices (Chung et al., 2017). Older adults show higher correlations between visual feedback and CMC (Watanabe et al., 2020).

Currently, few studies confirm that sensorimotor feedback alters Beta-CMC. Exploring CMC changes under various conditions using multimodal, multisensory stimuli may help to deepen our understanding of communication between the cerebral cortex and muscles during different motor processes, revealing the complexity of the motor system with its unique functional features.

## 5 The influencing factors of Beta band corticomuscular coherence

**Figure 4:**
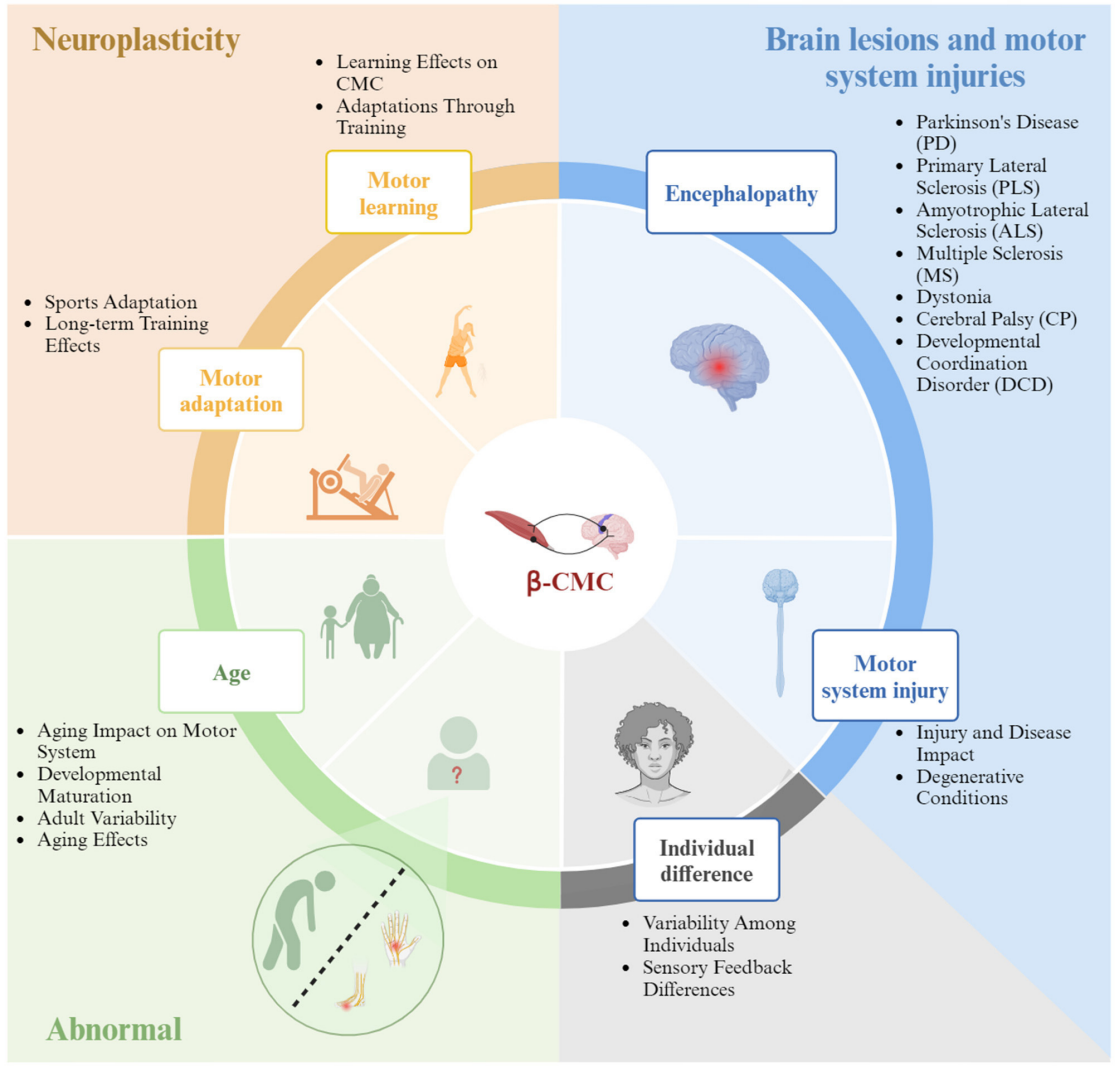
The influencing factors of Beta band corticomuscular coherence.

Research on Beta-CMC has significantly advanced our understanding, yet several key challenges remain in areas such as mechanistic insights, personalized interventions, long-term effects, and practical applications. Addressing these challenges requires leveraging advanced technologies, emphasizing individual differences, conducting long-term follow-up studies, and translating laboratory findings into clinical applications. By overcoming these challenges, we can deepen our understanding of CMC, develop new strategies for improving motor function and treating neurological disorders, and ultimately enhance both scientific research and patient health outcomes. Below is a summary of potential influencing factors on Beta-CMC, with a brief overview provided in Figure 4.

### 5.1 Age-related changes in Beta-band CMC

Age significantly impacts the motor system, with changes in Beta-CMC reflecting developmental and aging processes. During childhood, motor development relies on the formation and integration of neuronal networks within the sensorimotor system (Müller et al., 1991; Paus et al., 1999).

- Infants and children: Ritterband-Rosenbaum et al. (2017) observed significant increases in CMC within the 20–40 Hz frequency band between 9–25 weeks in infants, suggesting a sensitive period for corticospinal connection development.
- Adolescents and adults: beta-CMC increases with age, particularly around 20 Hz between ages 8–12 (James et al., 2008). Adults (20–30 years) exhibit higher CMC strength than children (8–10 years), primarily due to increased descending connections (Beck et al., 2021).
- Elderly: in the elderly, CMC increases under cognitive task conditions, but Beta-CMC declines in frequency while increasing in amplitude. However, older adults show a decline in M1's beta activity and CMC frequency, with an increase in amplitude (Johnson and Shinohara, 2012; Kamp et al., 2013). Bayram's et al. (2015) study showed significantly weakened CMC at all tested force levels in older adults.

Further research is needed to understand these age-related variations, particularly in older adults, to improve interventions aimed at mitigating motor decline associated with aging (Roeder et al., 2020; Yokoyama et al., 2020).

### 5.2 Individual differences in Beta-band CMC

Beta-CMC exhibits significant individual variability. Ushiyama et al. (2011) found substantial differences in the strength of oscillatory coupling between the motor cortex and spinal motor neurons among individuals.

- Force variability: the maximum value of CMC (CMC-max) positively correlates with the coefficient of variation of muscle force (Force-CV) and the power spectral density of muscle force output (Force-PSD) in various frequency bands.
- Types of contractions: during different types of muscle contractions (isometric, concentric, and eccentric), CMC and spinal excitability exhibit various changes (Glories et al., 2021; Glories and Duclay, 2023). Spinal inhibitory mechanisms may regulate Beta-band CMC, acting as a neural “filter” by modulating motor neuron activity (Williams and Baker, 2009; Williams et al., 2010; Matsuya et al., 2017). Sensory feedback variability and gain modulation at low and high beta frequencies also contribute to individual differences in CMC (Baker and Baker, 2003; Khademi et al., 2018).

### 5.3 Motor skill learning and control

Learning can enhance both CMC and motor performance. Méndez-Balbuena et al. (2012) showed that participants with and without pre-intervention CMC exhibited increases in CMC and motor performance after learning.

- Visuomotor skills: Perez et al. (2006) found that learning visuomotor skills increases Beta-CMC between cortical-spinal transmission and spinal motor neurons.
- Rhythmic patterns: learning to produce rhythmic musical patterns enhances corticomuscular communication (Lapenta et al., 2022).
- Strength training: 3 weeks of maximal strength training significantly increased muscle strength and improved motor coordination, associated with a reduction in antagonist muscle activation and CMC (Elie et al., 2021).

Exploring the impact of different exercise modalities on Beta-CMC can deepen our understanding of how exercise influences brain function and motor control.

### 5.4 Training status

Physical training engages multiple biological mechanisms, leading to significant changes in CMC and muscle coordination. Training status, especially in elite athletic groups, involves influences beyond motor skill learning. These influences include various behavioral and demographic factors that contribute to neurobiological differences. For example, elite athletes often have enhanced proprioception, muscle memory, and refined motor control, which are products of both intensive training and genetic predispositions.

- Athletes: long-term trained athletes like ballet dancers and weightlifters exhibit suppressed oscillatory coupling between the sensorimotor cortex and spinal motor neurons (Ushiyama et al., 2010). Strength trainers have the highest CMC strength and frequency, particularly in antagonist muscles (Dal Maso et al., 2017; Hortobágyi et al., 2021).
- Sports injuries: ACL injuries cause continuous imbalances in leg muscle strength. Patients with ACL reconstruction show decreased quadriceps strength and stability compared to uninjured controls (Sherman et al., 2023).

Similarly, the process of rehabilitation and the restoration of motor function post-injury can be seen as a specialized training status. This rehabilitation involves not just regaining lost strength and coordination but also adapting the brain-muscle communication to compensate for altered or damaged neural pathways. For instance, anterior cruciate ligament (ACL) injuries cause continuous imbalances in leg muscle strength, and patients with ACL reconstruction show decreased quadriceps strength and stability compared to uninjured controls. Standardizing participant selection and exploring CMC indices during sport adaptation can provide insights into the dynamic relationships between the brain and muscles post-injury.

### 5.5 The abnormal state of the Beta band CMC

Pain and muscle fatigue impact CMC and motor performance.

- Pain: both noxious and non-noxious sensory inputs modulate the functional coupling between the motor cortex and muscles. Pain reduces CMC, increases EEG frequency, and decreases force stability (Burns et al., 2016; Poortvliet et al., 2019).
- Fatigue: muscle fatigue leads to reduced information flow in descending pathways and weakens Beta-band brain-muscle signal coupling (Tecchio et al., 2006; Yang et al., 2009). Increased cortical drive may help maintain motor performance in fatigue states but could also exacerbate central fatigue (Gandevia, 2001).

Future research should aim to optimize neuromuscular interactions to improve fatigue management and recovery strategies. This includes enhancing CMC during fatigue through targeted training programs, neuromodulation techniques, and optimized recovery protocols. By focusing on the brain-muscle interplay under conditions of pain and fatigue, researchers can develop interventions to sustain motor performance and reduce injury risk, thereby enhancing athletic performance and well-being. Personalized approaches considering individual variability in pain perception and fatigue response can lead to more effective management strategies.

#### 5.5.1 Neurodegenerative diseases related to the Beta band of CMC changes

Understanding corticomuscular interactions in various states is crucial for diagnosing movement disorders and developing effective treatments. Neurodegenerative diseases, characterized by neuronal loss and disrupted glial cell homeostasis, often feature altered beta oscillations.

- Parkinson's disease (PD): PD patients exhibit excessive beta activity in basal ganglia circuits and reduced cortical beta activity due to dopaminergic neuron loss in the substantia nigra. This imbalance leads to enhanced and synchronized beta oscillations linked to motor dysfunctions (McCarthy et al., 2011; Little and Brown, 2014; Cole et al., 2017). Reduced CMC in PD patients correlate with motor symptom severity (Zokaei et al., 2021). Beta-CMC changes serve as biomarkers for PD, with increased low-frequency (~10 Hz) and decreased ~30 Hz CMC during stable contractions (McKeown et al., 2006). Levodopa modulates abnormal Beta-CMC, indicating its potential as a pathological marker (Hirschmann et al., 2013).
- Primary lateral sclerosis (PLS) and amyotrophic lateral sclerosis (ALS): in patients with primary lateral sclerosis (PLS), significant Beta-CMC was detected in the ipsilateral primary motor cortex (M1). PLS primarily affects upper motor neurons, whereas amyotrophic lateral sclerosis (ALS) impacts both upper and lower motor neurons. PLS patients exhibit significant differences in CMC across various frequency bands, which extend beyond primary sensory-motor networks (Bista et al., 2023). ALS patients show reduced CMC and increased cortical-cortical coherence, highlighting cortical network impairments (Proudfoot et al., 2018).
- Multiple sclerosis (MS): MS patients exhibit motor system disorders and higher CMC frequency without significant amplitude differences, linked to functional connectivity changes (Tomasevic et al., 2013).
- Dystonia: characterized by sustained muscle contractions and abnormal movements, dystonia shows aberrant Beta-CMC modulation, suggesting distinct sensory-motor processing abnormalities (McClelland et al., 2020). Sensory tricks can improve sensory-motor integration in dystonia (Lee et al., 2021).
- Cerebral palsy (CP): CP patients exhibit higher Beta-CMC compared to healthy controls, unaffected by measurement time windows (Riquelme et al., 2014). Muscle fatigue impacts CMC similarly in CP and neurotypical adults, but CP patients show baseline deficiencies in cortical-muscle coherence (Forman et al., 2022).

Despite the significant potential of Beta-band cortico-muscular coherence (Beta-CMC) in diagnosing and assessing treatment efficacy for neurodegenerative diseases, current research faces several challenges and limitations. In Parkinson's disease (PD), patients exhibit a marked imbalance in beta oscillations; while Levodopa can modulate Beta-CMC, it does not address the progressive neuronal loss. The complexity of CMC changes in primary lateral sclerosis (PLS) and amyotrophic lateral sclerosis (ALS) is not fully understood, complicating treatment strategies. Multiple sclerosis (MS) patients show elevated CMC frequencies, but the underlying mechanisms of these functional connectivity changes remain unclear. In dystonia, abnormal Beta-CMC modulation indicates sensory-motor processing abnormalities that require further investigation. Cerebral palsy (CP) patients exhibit elevated Beta-CMC levels, yet the baseline deficits in cortico-muscular coherence warrant additional exploration. Most studies are constrained by small sample sizes and specific experimental conditions, limiting the generalizability of the findings. Future research should aim to validate these results in larger, more diverse populations and focus on long-term neuroplasticity and functional recovery mechanisms through longitudinal studies. Relying solely on Beta-CMC measurements may not fully capture the complex neurophysiological processes; integrating multiple biomarkers could provide a more comprehensive assessment. Moreover, the clinical application faces challenges such as device portability, ease of use, and real-time data analysis. Thus, developing user-friendly and reliable measurement and analysis tools is essential to advance the clinical utility of Beta-CMC.

#### 5.5.2 Changes in Beta-band CMC resulting from motor system injuries

Motor system injuries, such as those from sports and strokes, significantly impact CMC and brain-muscle communication.

- Sports injuries: repetitive impacts, such as heading in soccer, can cause brain injuries. Studies show enhanced Beta-CMC in real environments but not in VR, possibly due to sensory input differences (Parr et al., 2023b). This compensatory mechanism may indicate a risk of long-term brain injury while demonstrating the brain's adaptive strategies (Campus et al., 2012; Chipaux et al., 2013). While such adaptations could indicate a risk of long-term brain injury, they also demonstrate the brain's strategy to cope with challenges.
- Stroke: stroke-induced motor impairments are linked to brain network reorganization. Stroke patients show widespread CMC peaks, including contralateral hemisphere peaks (Rossiter et al., 2013; Krauth et al., 2019). Changes in CMC correlate more with post-stroke duration than with motor recovery degree (von Carlowitz-Ghori et al., 2014). Motor performance improvement post-stroke is associated with increased Beta-CMC over time (Larsen et al., 2017). Another study reported that as motor ability gradually recovered post-stroke, Beta-CMC increased over time, surpassing levels seen in healthy controls (Krauth et al., 2019).
- Spinal cord injuries (SCI): SCI patients exhibit higher muscle co-activation and lower frequency CMC, particularly in intermuscular coupling (Zu et al., 2023). Despite unchanged cortical efficacy, SCI patients increase muscle activation to compensate for reduced cortico-muscular communication (Cremoux et al., 2013).

In neurobiology, changes in beta-band cortico-muscular coherence (Beta-CMC) resulting from motor system injuries exhibit several commonalities. The brain demonstrates significant adaptive mechanisms to cope with injuries such as sports injuries, strokes, and spinal cord injuries, reorganizing brain-muscle communication pathways. For instance, enhanced Beta-CMC in real environments for sports injuries indicates adaptation to sensory inputs, while in stroke patients, Beta-CMC increases over time, reflecting cortical reorganization. Similarly, spinal cord injury patients compensate for reduced cortico-muscular communication by increasing muscle activation. Furthermore, Beta-CMC serves as a potential biomarker for recovery and adaptation. In stroke patients, Beta-CMC progressively increases with motor recovery, eventually surpassing healthy controls. Changes in Beta-CMC in sports and spinal cord injury patients also reflect their adaptive mechanisms, aiding in assessing rehabilitation progress and designing personalized strategies. These commonalities provide insights into the functional connectivity between the brain and muscles post-injury and highlight Beta-CMC's potential as a biomarker for guiding rehabilitation therapies.

Although beta-band cortico-muscular coherence (Beta-CMC) shows potential in assessing recovery following motor system injuries, several limitations remain. Current research predominantly involves small sample sizes and specific experimental conditions, which may limit the generalizability of findings. Future studies should aim to validate these results across larger and more diverse patient populations. Additionally, while existing studies primarily focus on short-term recovery, there is a significant gap in understanding the mechanisms underlying long-term neuroplasticity and functional recovery. Longitudinal studies are needed to address this gap. Moreover, Beta-CMC as a solitary biomarker may not adequately capture the multifaceted processes involved in motor function recovery. Integrating multiple biomarkers could provide a more comprehensive assessment. Furthermore, the clinical application of Beta-CMC faces practical challenges, including the portability of measurement devices, ease of operation, and real-time data analysis. To facilitate its clinical use, it is essential to develop user-friendly and reliable measurement and analysis tools. Therefore, despite its promise, future research should prioritize expanding sample sizes, investigating long-term effects, combining multiple biomarkers, and developing practical clinical tools to advance this field.

## 6 Beta band of CMC in clinical rehabilitation, and the application prospect in the field of competitive sports

Beta-CMC has significant potential to revolutionize clinical rehabilitation and enhance performance in competitive sports. Techniques like transcranial Direct Current Stimulation (tDCS), transcranial Alternating Current Stimulation (tACS), and Neuromuscular Electrical Stimulation (NMES) have shown promising results in enhancing CMC, thereby improving motor function and aiding in recovery from conditions such as stroke and multiple sclerosis (Bao et al., 2019; Padalino et al., 2021; Kudo et al., 2022). However, the effects of these techniques can vary significantly among individuals, indicating a need for personalized approaches (Schilberg et al., 2018; Ibáñez et al., 2023). Personalized approaches should consider factors such as individual neurophysiological profiles, optimal stimulation parameters, and the integration of multimodal feedback systems.

In competitive sports, understanding and optimizing Beta-CMC can provide critical insights into fatigue management, injury prevention, and skill refinement, leading to superior athletic performance. Future research should focus on the specific impacts of different exercise modalities and intensities on CMC (Pan et al., 2018; Xu et al., 2018; Koseki et al., 2021). Studies should also explore the interplay between CMC and various forms of athletic training to determine the most effective methods for enhancing performance. Advancements in non-invasive brain stimulation and neuroimaging techniques are expected to further our understanding of Beta-CMC mechanisms, facilitating personalized rehabilitation strategies tailored to individual neural dynamics. Interdisciplinary research is crucial to fully leverage the potential of Beta-CMC in both clinical and athletic contexts, refining current practices and developing innovative approaches for enhancing motor function and recovery. See Table 2 for a summary of changes in Beta-band CMC in clinical rehabilitation and competitive sports.

**Table 2:** Changes in Beta-band CMC in clinical rehabilitation and competitive sports.

**Application area**	**Findings and changes**	**References**
**Clinical rehabilitation**		
tDCS and tACmS	Immediate enhancements in CMC and MEPs, particularly in stroke and MS patients, showing greater recovery effects	Bao et al., 2019; Padalino et al., 2021; Kudo et al., 2022
Individual differences	Effects of tACS on CMC vary among individuals; 20 Hz tACS modulates MEP amplitude in some studies but has unclear effects on Beta-CMC	Schilberg et al., 2018; Ibáñez et al., 2023
NMES	NMES at beta frequencies impacts CMC and voluntary motor output correlation; combined with exercise training shows significant rehabilitation effects.	Pan et al., 2018; Xu et al., 2018; Koseki et al., 2021
BCI and neurofeedback	Enhances Beta-CMC control, especially in chronic stroke patients; real-time CMC feedback training improves motor function.	von Carlowitz-Ghori et al., 2015; Belardinelli et al., 2017; Khan et al., 2020; Khademi et al., 2022
**Competitive sports**	**Future direction**	
Fatigue management	Optimizing CMC provides critical insights into fatigue management, injury prevention, and skill refinement	Søgaard et al., 2006; Yang, 2008; Yang et al., 2009; Tomasevic et al., 2013; Liang et al., 2021
Athletic performance	Specific impacts of different exercise modalities and intensities on CMC need further study	Dal Maso et al., 2012, 2017
Personalized training	Understanding and optimizing CMC can lead to superior athletic performance and training outcomes	Ushiyama et al., 2010; Elie et al., 2021

## 7 Future directions

This review underscores the integral role of Beta-CMC in advancing motor control. Future studies should explore individualized neuromodulation strategies, incorporating real-time neurofeedback to optimize CMC modulation based on personal neurophysiological profiles (Ding et al., 2023). By understanding the mechanistic basis of Beta-CMC across different motor tasks and its modulation via neuromodulation techniques, personalized medicine approaches can be developed to customize interventions according to individual cortical rhythms and motor profiles. Addressing the variability and dynamics of Beta-CMC through targeted research will enhance the efficacy of therapeutic interventions and athletic training programs.

### 7.1 Individual variability in CMC responses

Existing research indicates significant variability in the effectiveness of personalized approaches, which is likely attributable to differences in individual neurophysiological characteristics. However, the specific mechanisms underlying these differences remain unclear. Investigating these mechanisms is crucial for optimizing personalized treatment strategies and improving outcomes. Below are some rigorous examples that discuss the causes of individual differences:

1. Neuroanatomical differences:
  - Case study: Schilberg et al. (2018) found significant differences in the effects of tACS among individuals, potentially related to neuroanatomical variations. Some individuals exhibit stronger neuronal synchronization at specific frequencies, while others do not. These differences may stem from factors such as cortical thickness and gray matter density.
2. Neurophysiological state:
  - Experimental study: Ibáñez et al. (2023) found that baseline neural activity levels significantly influence the effectiveness of tDCS. Individuals with higher baseline neural activity levels showed more pronounced improvements in CMC with the same stimulation intensity. This indicates that the neurophysiological state of an individual is a crucial factor affecting treatment outcomes.
3. Individualized neurofeedback systems:
  - Empirical evidence: Koseki et al. (2021) demonstrated that sensory inputs based on individual CMC frequencies significantly affect the relationship between CMC and voluntary motor output. This suggests that personalized sensory feedback systems are essential for optimizing treatment outcomes.
4. Long-term adaptive changes:
  - Longitudinal study: Xu et al. (2018) found that long-term exercise training combined with sensory stimulation significantly enhances CMC, with substantial individual differences. This indicates that athletes experience adaptive changes in their nervous systems over time, reflecting different adaptive mechanisms among individuals.

Investigating these individual differences will provide important references for optimizing personalized treatment strategies, thereby improving therapeutic outcomes and athletic performance.

## 8 Summary

Beta-band corticomuscular coherence holds significant potential in clinical rehabilitation and competitive sports. Future research should prioritize exploring individualized neuromodulation strategies, incorporating real-time neurofeedback to optimize CMC modulation based on personal neurophysiological profiles. By understanding the mechanistic basis of Beta-CMC across different motor tasks and its modulation through neuromodulation techniques, personalized medicine approaches can be developed to customize interventions according to individual cortical rhythms and motor profiles. This personalized approach could significantly improve therapeutic outcomes and athletic performance by addressing the unique needs of each individual.

## Author contributions

JP: Writing – original draft, Writing – review & editing. TZ: Writing – original draft, Writing – review & editing. ZS: Investigation, Writing – original draft, Methodology. KS: Writing – review & editing.
